# FUTURE SELF CONTINUITY AND AGING PREPARATION ACROSS CULTURES: THE MODERATING ROLE OF FUTURE SELF-VIEWS

**DOI:** 10.1093/geroni/igae098.1734

**Published:** 2024-12-31

**Authors:** Yiwen Wu, Helene H Fung

**Affiliations:** The Chinese University of Hong Kong, Hong Kong, China (People’s Republic); The Chinese University of Hong Kong, Hong Kong, China (People’s Republic)

## Abstract

Preparation for aging enhances well-being and lowers societal costs. According to Hershfield (2019), people view their future selves as strangers when making intertemporal decisions. Future self-continuity—the psychological connectedness to future selves—is crucial to people’s willingness to allocate resources for the future (Hershfield et. al., 2011). While studies have shown that higher future self-continuity promotes saving intentions (e.g. Hershfield et al., 2020), such relationships have not been examined among older populations in diverse cultures. This research aims to investigate the effect of future self-continuity on aging preparation and the moderating role of future self-views in two studies across six cultures. Two separate surveys have been conducted on N = 117 Australian older adults aged 55-86 years (Study 1, Mage = 61.7 SD = 6.6) and N = 2,940 older adults aged 38-99 years (Study 2, Mage = 61.5 SD = 15.2) across five cultures (Hong Kong, Taiwan, Germany, Czechia, and the United States). The results showed that future self-continuity predicted more preparation for aging in both studies, after controlling for age, gender, health, and social status. Further, the effect of future self-continuity on aging preparation was conditional on the valence of future self-views. Specifically, future self-continuity was positively associated with aging preparation when people held positive views towards their future selves, while such relationships became negative when people viewed their future selves negatively. These findings highlight the importance of considering both future self-continuity and future self-views in interventions to promote aging preparation among older adults across diverse cultures.

